# Sudden Appearance of a Palpable Chest Wall Mass Secondary to Macrocystic Lymphatic Malformation: A Case Report

**DOI:** 10.3390/children10020235

**Published:** 2023-01-28

**Authors:** Hend Alkwai, Hala Alkwai, Mohammed Al Namshan

**Affiliations:** 1 Department of Pediatrics, College of Medicine, University of Ha’il, Hail 55255, Saudi Arabia; 2 Department of Pulmonology, King Abdullah Specialized Children’s Hospital, Riyadh 14611, Saudi Arabia; 3 Department of Pediatric Surgery, King Abdullah Specialized Children’s Hospital, Riyadh 14611, Saudi Arabia

**Keywords:** case report, lymphatic malformation, macrocystic lymphatic malformation, lymphangioma, cystic hygroma, chest wall

## Abstract

Chest wall lymphatic malformations are rare and can pose a diagnostic dilemma, particularly if they present abruptly. This case report describes a 15-month-old male toddler presenting with a left lateral chest mass. Histopathology of the surgically excised mass confirmed the diagnosis of a macrocystic lymphatic malformation. Furthermore, there was no recurrence of the lesion in the two-year follow-up period.

## 1. Introduction

In children, palpable chest wall masses occur due to variable underlying pathologies, including congenital malformations, infections, and benign and malignant tumours. A biopsy and histopathological examination aid in the definitive diagnosis.

Lymphatic malformations (LMs) are vascular anomalies composed of abnormal, dilated lymphatic channels caused by aberrant development. According to the updated classification of the International Society for the Study of Vascular Anomalies (ISSVA), LMs are considered low-flow, simple vascular malformations [1]. The common (cystic) lymphatic malformation type is further divided into three subtypes according to the size of the cysts. Macrocystic LMs (mLMs) are rare [2,3], and those involving the chest wall are even rarer and can present a diagnostic dilemma, particularly if they appear suddenly. Here, we describe the clinical, radiological, and pathological findings of a lateral chest wall macrocystic lymphatic malformation presenting acutely in a 15-month-old male and managed by surgical excision with no recurrence in the two-year follow-up post-excision.

## 2. Case Report

A 15-month-old, previously well, male toddler presented to the general paediatric clinic with a rounded mass involving the left lateral chest wall noticed over the last two weeks. The mass was first noticed by his mother during bedtime while changing him into his sleepwear. It seems to have appeared suddenly, with no history of previous trauma or recent infections. Additionally, while the mass did not seem to cause pain, there was discomfort, especially when in close contact with clothing. Although the size of the mass did not change since its appearance, the overlying skin developed a red/blue hue over the next few days after its appearance. There was no history of other similar masses in the patient or his family.

**Figure 1:**
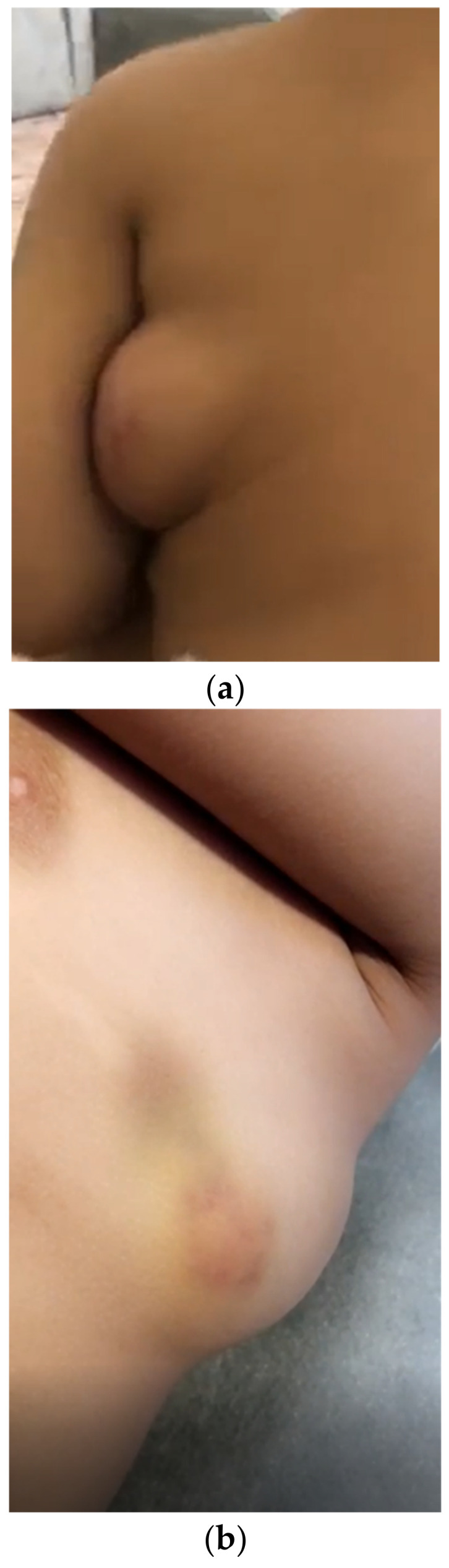
(**a**,**b**): The mass over the left lateral chest (**a**) posterior and (**b**) anterior images.

On examination, there was a single, oval-shaped mass measuring around 6 × 4 × 2 cm located on the left lateral chest wall (Figure 1a,b). The mass was mobile, non-tender, soft, and compressible. The overlying skin had a red/blue hue. The mass was attached to the overlying skin but not to the underlying structures. There was positive transillumination, and on auscultation there was no bruit. The rest of the physical examination was unremarkable.

**Figure 2:**
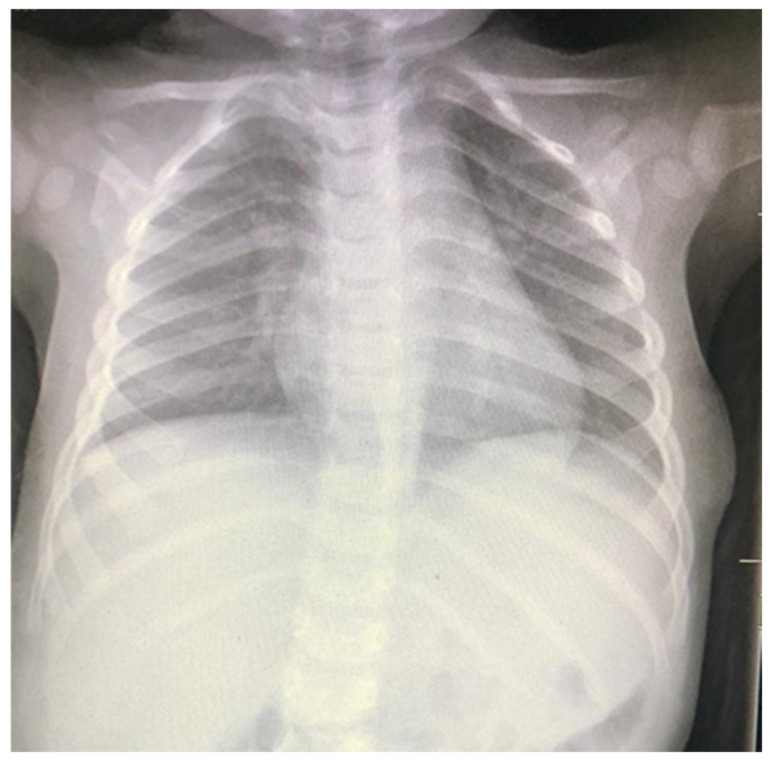
Chest X-ray showing the soft tissue mass with intact ribs.

**Figure 3:**
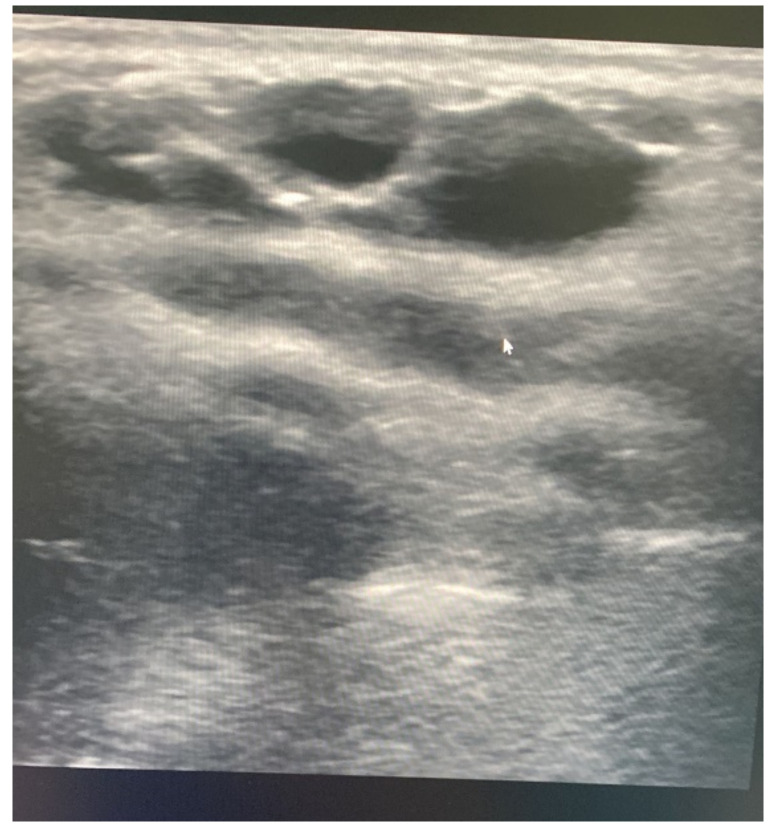
Transverse grey-scale ultrasound image shows an anechoic multilocular cystic lesion in the left lateral chest wall.

Basic laboratory investigations, including a complete blood count with a differential count, were normal. Chest radiography revealed a mass-like opacity in the left lateral chest wall (Figure 2). An ultrasound scan (USS) of the chest wall detected multiple, well-circumscribed, anechoic subcutaneous cystic lesions of variable size, the largest measuring 2.1 × 0.7 cm (Figure 3). Colour Doppler USS showed no vascularity within the lesions. A chest computed tomography (CT) was requested to assess the mass and its relation to adjacent structures. Axial and coronal CT images revealed low-attenuated, thin-walled lesions overlying ribs six through nine. There was no evidence of internal calcifications with clear surrounding fat planes. The mass was indenting the anterior surface of the serratus anterior muscle. Nevertheless, there was a clear line of demarcation between the mass and the underlying muscle. There was no evidence of intrathoracic or intra-abdominal extension. Additionally, there was no enhancement post-intravenous contrast. The USS was sufficient to make a preliminary diagnosis. To assess for extension, a CT scan was preferred over magnetic resonance imaging (MRI) in our patient due to the shorter duration of the procedure and the lack of need for sedation.

**Figure 4:**
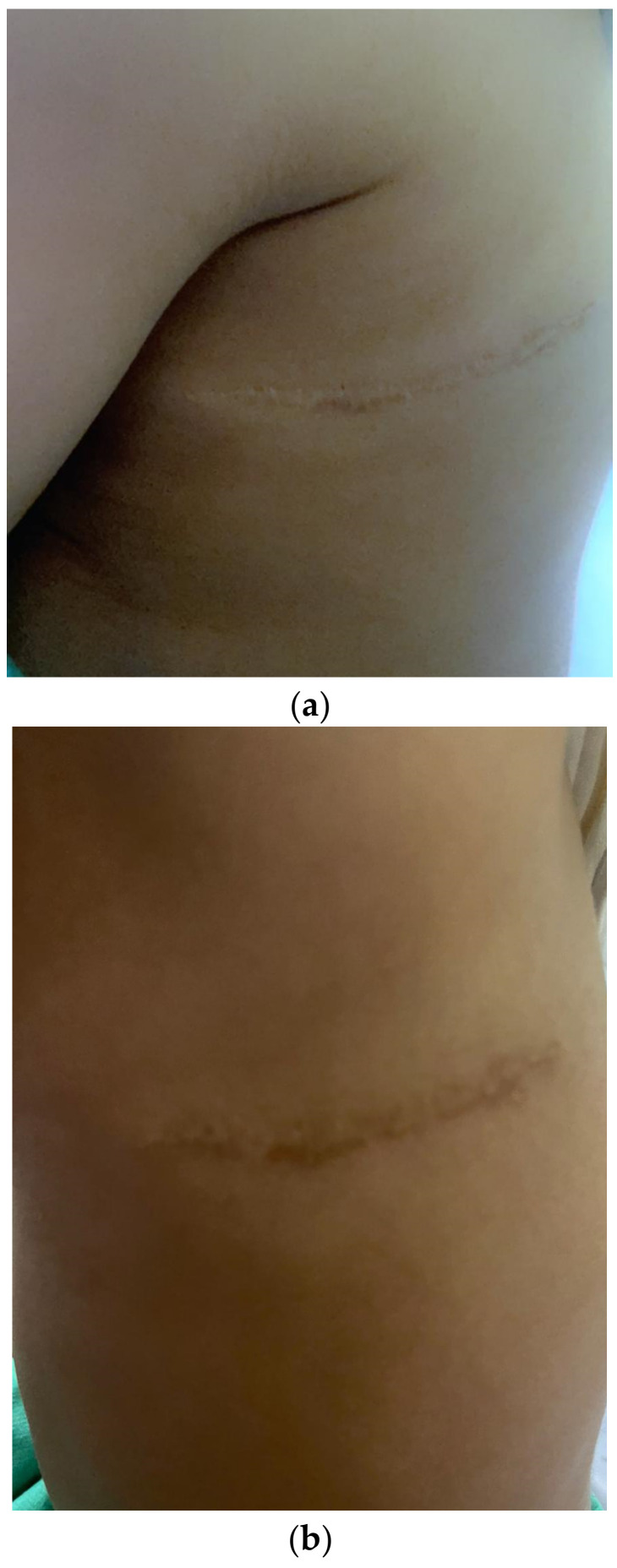
(**a**,**b**): Incision scar with no local recurrence after follow-up.

The preliminary diagnosis was that of a lymphatic malformation, and the patient was referred to a tertiary hospital for further management. A thorough discussion of three different treatment modalities, namely expectant management, sclerotherapy, and surgery was had with the family. Although the lesion was small, well-localised, and not impinging on vital structures, expectant management was dismissed as the pre-verbal patient seemed preoccupied with the mass, persistently pulling on the lesion, and voicing sounds suggestive of discomfort. As for sclerotherapy, the patient would require sedation and possibly general anaesthesia. Furthermore, repeat sclerotherapy might be warranted in the event of incomplete resolution. Based on imaging, total resection of the mass was likely feasible. To that end, possible complications with surgery and the remote risk of recurrence were explained in detail. Upon further discussion, the parents shared their preoccupation with a diagnosis of a malignant mass. Conclusively, a decision for surgery was made. In the operating room and under general anaesthesia, an incision was made over the lesion. Circumferential dissection was made using both blunt and sharp dissection. The lesion was found to be attached to the fascia of the underlying latissimus dorsi and overlying skin. The fascia of the muscle was excised, and the skin was trimmed. The subcutaneous tissue was approximated, and the wound was closed using absorbable sutures. Finally, surgical tape strips and a pressure dressing were applied. The patient tolerated the procedure well, with no postoperative complications. The postoperative histopathology report described dilated lymphatic channels lined by endothelial cells. Histopathology was compatible with a diagnosis of a macrocystic lymphatic malformation. The patient has had no recurrence of the lesion in the two years of follow-up post-excision (Figure 4a,b).

## 3. Discussion

The International Society for the Study of Vascular Anomalies (ISSVA) has classified vascular anomalies into five broad categories: vascular tumours, simple vascular malformations, combined vascular malformations, anomalies of major named vessels, and vascular malformations associated with other anomalies [1]. This classification was last revised in 2018 and is expected to evolve as new insights into the pathogenesis and genetics of vascular anomalies emerge.

Simple vascular malformations are mostly composed of one vessel type, such as veins, lymphatics, or capillaries. An exception is arteriovenous malformations, which are composed of several vessel types (different from combined vascular malformations, which are combinations of different distinct, simple vascular malformations) and the non-acquired arteriovenous fistula. Simple vascular malformations are subdivided according to the velocity of the flow. Low-flow lesions include venous malformations, lymphatic malformations, and capillary malformations. On the other hand, high-flow lesions include arteriovenous malformations and non-acquired arteriovenous fistulae. Collectively, they occur during morphogenesis, and unlike vascular tumours, the turnover of the endothelium is normal.

Lymphatic malformations (LMs) are low-flow lesions comprised of dilated lymphatic cysts or channels. They can occur in isolation or be combined with other vessel malformations. Failure of the lymphatic system to separate from, or connect with, the venous system during embryogenesis is thought to be central to their pathogenesis [4]. Recently, however, sporadic genetic abnormalities have been proposed [5]. Histologically, they are lined by endothelial cells. Hence, antibodies to lymphatic endothelial cells, such as Prospero-related homeobox gene-1 (Prox-1) and vascular endothelial growth factor receptor-3 (VEGFR-3), are used for the differentiation of LMs from venous malformations in pathological specimens [6]. Isolated LMs have been associated with somatic activating variants in PIK3CA [7] and, more recently, in BRAF [8].

The common or cystic lymphatic malformations (cLMs) are classified according to cyst size: macrocystic, microcystic, or mixed. However, there is no definite consensus regarding the defining size of the cysts [9]. Some classify lesions as macrocystic if they are larger than two centimetres in diameter, microcystic if they are less than two centimetres in diameter, or mixed if they contain cysts of both sizes [10]. Others use one centimetre as a cut-off size for differentiating macrocystic from microcystic LMs [11,12]. In the past, overlapping terminologies have been used to describe cLMs, such as lymphoceles, lymphangiomas, cavernous lymphangiomas, cystic lymphangiomas, cystic hygromas, and hygroma (colli) cysticum.

Collectively, cLMs comprise a group of rare, non-malignant diseases. Their prevalence is variable but is approximated at around 1 in 4000 live births with equal sex distribution [3]. They present primarily at birth or during the first two years of life. Some cLMS can be detected antenatally [13]. Nevertheless, LMs have been reported in all age groups. They have a predilection for lymphatic-rich areas such as the head, neck, and axilla but have been reported to involve different body parts. The size of LMs is variable, and while their growth is proportional to body growth, sudden enlargement can occur with an intracystic haemorrhage, trauma, or infection. The symptoms of cLMs depend on their location and size. Their presentation can vary from asymptomatic swelling to severe swelling, leading to disfigurement or life-threatening complications such as airway obstruction. Ultrasound scanning with Doppler is the initial imaging modality of choice [14]. MRI is used to assess the extension of the lesion; however, a CT scan can be preferred due to the shorter duration of the procedure and the lack of need for sedation, as is the case in our patient.

The treatment of LMs warrants a multidisciplinary approach. The choice of treatment and its urgency are individualised and depend on many factors, including the size of the lesion, its location, the presence of symptoms or functional impairment(s), available resources, the level of expertise, and patient preference. Expectant management is an option. Spontaneous regression is more likely to occur in cLMs with fewer than five septations, particularly in the posterior neck region [15,16]. Medical treatment to reduce swelling with corticosteroids, treat infections with antibiotics, or manage pain with analgesia can be used according to need. Trials assessing the effectiveness of targeted medical interventions have been prompted by the recognition of specific activating mutations in known oncogenic signalling pathways [17]. Systemic sirolimus has been associated with a reduction in size and a decrease in the rate of complications related to cLMs [18,19]. However, reported side effects, including mouth sores, gastrointestinal upset, bone marrow suppression, and alterations in laboratory parameters, necessitate monitoring drug levels and frequent clinical and laboratory follow-ups. Everolimus can be used as an alternative to sirolimus [20]. Sclerotherapy under image guidance is another treatment option. Various sclerosing agents have been used as well as combination injections of bleomycin with doxycycline [21]. Side effects from sclerotherapy depend on the sclerosant agent involved. Surgical resection is a potential cure, assuming that a complete resection is performed. Laser ablation therapy and virtual reality have been used as adjuvants to surgery [22]. However, surgery is not without risks. These are related to the anatomic site of the lesion, and its proximity to vital structures, in addition to the inherent risks of any surgical procedure. Recurrence rates of up to 40% have been reported [23]. All of these are taken into consideration when discussing treatment options with patients and their families.

In this report, we have presented a case of a macrocystic lymphatic malformation presenting peculiarly. The abrupt appearance of a left lateral chest wall in this toddler with no history of trauma or previous infection has presented a diagnostic dilemma to the primary healthcare provider. This has further increased parental anxiety. The simple bedside examination procedure as well as the typical findings on USS and CT were sufficient to make a preliminary diagnosis of lymphatic malformation. After discussing the advantages and disadvantages of the different treatment modalities, the choice of a complete surgical excision was made. The patient has had no recurrence in the two-year follow-up period; nevertheless, a longer follow-up is warranted as recurrence has been reported 20 years post-surgical excision [24]. Lymphatic malformations have been frequently described in the literature. However, few case reports describe isolated chest wall lymphatic malformations with no axillary or cervical extensions presenting outside the neonatal period [25,26,27].

## 4. Conclusions

Chest wall palpable masses encompass a variety of pathologies, including congenital malformations, infections, and benign and malignant tumours. Congenital causes should be kept in mind even with the sudden appearance of a mass, as trauma or infections can herald their appearance. Macrocystic LMs are one of these differential diagnoses. Newer treatment modalities have been used to manage LMs. Surgical excision is still a valid treatment option. However, recurrence is a possibility, especially after incomplete resection [24].

## Data Availability

Not applicable.
